# Efficacy of ginger on chemotherapy-induced nausea and vomiting in breast cancer: A meta-analysis

**DOI:** 10.1097/MD.0000000000051071

**Published:** 2026-10-30

**Authors:** Yanyan Tian, Mingde Ma, Zhengye Wang

**Affiliations:** a Department of Thyroid and Breast Surgery, Huaihe Hospital of Henan University, Kaifeng, Henan, China.

**Keywords:** breast cancer, chemotherapy, chemotherapy-induced nausea and vomiting, ginger, meta-analysis, *Zingiber officinale*

## Abstract

**Background:**

It is unclear that the efficacy of ginger in breast cancer (BC) patients with chemotherapy-induced nausea and vomiting (CINV).

**Methods:**

An extensive search of 4 databases Web of Science, Embase, PubMed, and Cochrane Library was conducted for clinical trials evaluating ginger in the treatment of CINV in BC patients, covering literature up to July 24, 2024. Outcome measures included the effect on scores, or the number of events related to nausea and vomiting, as well as scores of acute nausea, delayed nausea, and improvements in the nutritional status of BC patients. For the screened studies, Excel 2010 was used to extract key study information and specific data for each outcome indicator. Review Manager 5.4 was employed for the risk of bias assessment, and meta-analysis of the extracted data from the included studies was performed using Stata software.

**Results:**

A total of 9 randomized controlled trials were included. The risk of bias assessment indicated that most studies were of moderate quality. The meta-analysis results showed no significant difference in the reduction in the number of nausea events, nausea scores, acute nausea scores, delayed nausea scores, number of vomiting events, or vomiting scores between the ginger group and the control group using antiemetic drugs or placebo. However, ginger demonstrated a favorable safety profile in terms of adverse effects.

**Conclusion:**

Ginger appears to be safe for BC patients; however, its inhibitory effect on CINV remains inconclusive. Due to the limited number of available studies, accurate conclusions regarding the effectiveness of ginger in managing CINV in BC patients cannot be drawn. More rigorously designed, large-sample, high-quality randomized controlled trials are needed to validate the specific effects of ginger in this patient population.

## 1. Introduction

Breast cancer (BC) is the most commonly diagnosed malignancy among women worldwide, and chemotherapy remains an important component of treatment across different stages of the disease. Chemotherapy-induced nausea and vomiting (CINV) is among the most distressing treatment-related adverse effects and can negatively affect nutritional status, hydration, quality of life, and adherence to chemotherapy.^[1,2]^ With the gradual increase in awareness of BC and the standardization of various clinical guidelines, the prognostic status and quality of survival of BC patients have improved compared to the past. However, there is still a lack of awareness of screening for this disease in some populations; therefore, many patients have already developed intermediate and advanced stages by the time of their visit to the clinic, and this has made the treatment of the disease somewhat difficult. Similar to other cancers, chemotherapy is widely used in the treatment of BC patients, and the National Comprehensive Cancer Network guidelines for BC state that preoperative chemotherapy may be used in patients with locally advanced BC and patients with inoperable BC, such as patients with inflammatory BC and cT4 tumors.^[3]^

CINV remain one of the most distressing and common side effects in BC treatment. Studies have shown that up to 80% of patients receiving chemotherapy experience CINV, which can lead to dehydration, malnutrition, reduced quality of life, and decreased treatment adherence. Despite the use of various antiemetic medications, including 5-hydroxytryptamine type 3 (5-HT3) receptor antagonists, neurokinin-1 receptor antagonists, and corticosteroids, some patients continue to experience inadequate control of CINV, many patients still suffer from inadequate symptom relief or intolerable side effects. There is growing interest in complementary approaches to manage CINV, and ginger (Zingiber officinale) has long been used in traditional medicine for its antiemetic properties. Ginger contains active compounds, such as gingerols and shogaols, which may inhibit CINV by modulating serotonin receptors and reducing gastrointestinal irritation. While several studies have suggested that ginger may be effective in reducing CINV, its role in patients with BC undergoing chemotherapy has not been fully clarified. Therefore, this meta-analysis aims to evaluate the efficacy and safety of ginger in managing CINV specifically in BC patients, providing updated evidence for its potential integration into supportive cancer care.

The risk and severity of CINV are influenced by the emetogenic potential of the chemotherapy regimen and individual patient-related factors. Doxorubicin + cyclophosphamide (AC) and epirubicin + cyclophosphamide regimens are dominated by anthracyclines, such as doxorubicin and epirubicin. Docetaxel + doxorubicin + cyclophosphamide (TAC) protocol combines anthracycline and taxanes, such as docetaxel and paclitaxel. The doxorubicin + cyclophosphamide followed by a taxane scheme was the sequential application scheme for anthracyclines and taxanes. The docetaxel + cyclophosphamide regimen is an anthracycline-free regimen, such as docetaxel and cyclophosphamide, and the other regimen, such as capecitabine regimen, for patients with triple-negative BC.^[4]^ Although these chemotherapeutic agents can inhibit tumor cells and reduce the risk of recurrence and death in early-stage BC, they bring with them side effects of chemotherapy, such as nausea and vomiting, liver and kidney damage, and neurotoxic reactions, which are usually difficult to avoid.^[5]^ Studies have found that 60% to 80% of chemotherapy patients will experience CINV, which is one of the most common adverse reactions.^[6,7]^ If the control of CINV is not ideal or difficult to control, it will lead to poor nutritional status, water and electrolyte disorders, and other manifestations in patients, which will not only affect the treatment of BC, but also have a serious impact on their quality of life, and even cause fatal injuries to BC patients.^[8,9]^ Chemotherapeutic agents are classified into 4 classes according to their CINV risk, with cyclophosphamide having a high emetogenic risk and anthracyclines having a moderate emetogenic risk.^[10]^

Current prevention and treatment of CINV mainly rely on combinations of 5-hydroxytryptamine-3 (5-HT3) receptor antagonists, neurokinin-1 receptor antagonists, corticosteroids, and other antiemetic agents. Although these treatments have substantially improved CINV control, some patients continue to experience acute or delayed nausea and vomiting despite appropriate prophylaxis. In addition, treatment-related adverse effects and incomplete symptom control have encouraged interest in safe complementary approaches, but they are still associated with varying degrees of side effects such as headache, constipation, and liver damage.^[11]^ How to safely and effectively prevent CINV due to chemotherapy in BC patients, improve patients’ quality of life during chemotherapy, and enhance patients’ adherence to chemotherapy are current challenges that need to be addressed. Medicinal plants and their bioactive constituents have attracted increasing interest because of their diverse pharmacological and therapeutic properties. Recent investigations of plant-derived compounds have demonstrated antioxidant, anti-inflammatory, anticancer, and other biological activities, supporting continued research into natural products as complementary therapeutic approaches.^[12,13]^ Ginger is of particular interest in CINV because its major bioactive constituents, including gingerols and shogaols, have demonstrated antiemetic-related activity, particularly through modulation of serotonergic pathways such as 5-hydroxytryptamine type 3 receptor (5-HT3) receptor signaling.. Ginger contains active ingredients such as curcuminoids, volatile oils, and polysaccharides. Its main functional substances include gingerols, paradol, and gingersol. These compounds have been found to possess a wide range of pharmacological activities such as anti-inflammatory antioxidant, anticancer, neuroprotective, and most importantly, they can be used for anti-CINV.^[14]^ It has been found that gingerols in ginger can inhibit 5-HT3 receptors to improve nausea and vomiting, while gingerols and thiols can bind to the corresponding targets through the ion channels of 5-HT3 receptors to exert antiemetic effects.^[15]^ Several clinical trials have investigated ginger for the management of CINV, with some reporting potential benefits; however, the overall findings remain inconsistent, and the available evidence does not establish a consistent significant effect or the complete absence of adverse effects.^[16]^ Because of the advantages that ginger has shown for CINV, we wanted to investigate the efficacy of ginger in inhibiting CINV when applied to BC patients undergoing chemotherapy. Several clinical trials and systematic reviews have evaluated ginger for CINV, but their findings remain inconsistent, potentially because of differences in cancer populations, ginger dosage and formulation, chemotherapy regimens, concomitant antiemetic treatment, and methodological quality. Importantly, previous evidence has generally included heterogeneous cancer populations, while the efficacy and safety of ginger specifically in BC patients remain insufficiently defined. Therefore, this systematic review and meta-analysis aimed to evaluate the efficacy and safety of ginger for preventing or reducing CINV specifically in patients with BC. However, an important unresolved issue is dose dependency: some studies suggest that low to moderate doses of ginger (e.g., ≤1 g/day) may be insufficient to achieve therapeutic effect, while higher doses may pose gastrointestinal side effects. Additionally, the formulation of ginger (capsules, extracts, essential oils, and food-based preparations) may influence its pharmacological activity and bioavailability. For instance, encapsulated ginger powder may have delayed absorption compared to liquid extracts or sublingual preparations. The placebo effect is also a notable factor in CINV research, given the subjective nature of symptom reporting and high expectations of complementary therapies. These methodological variations may partly explain the conflicting results among randomized controlled trials (RCTs). Therefore, a focused meta-analysis on BC patients, considering these challenges, is needed to clarify ginger’s potential benefits and limitations in this specific clinical setting.^[17]^ Therefore, this study systematically searched for clinical trials on the use of ginger during chemotherapy in BC patients in recent years to investigate the various effects of ginger on the production of BC chemotherapy, as well as its efficacy and safety against CINV, and to make a study and discussion on the results to provide relevant evidence-based evidence for the use of ginger in BC chemotherapy patients.

While several studies and meta-analyses have explored the role of ginger in alleviating CINV across various cancers, few have focused specifically on BC patients: a group that commonly receives anthracycline- and taxane-based chemotherapy, which carry moderate to high emetogenic risk. Moreover, BC remains the most common malignancy among women globally, and patients undergoing chemotherapy frequently report persistent nausea and vomiting, despite the use of standard antiemetics. Therefore, evaluating the effect of ginger specifically in this population is clinically relevant and necessary.

The aim of this meta-analysis is to systematically assess the efficacy and safety of ginger as a complementary treatment for CINV in BC patients, providing targeted evidence for supportive care strategies in this widely affected group.

## 2. Materials and methods

### 2.1. Data sources and searching strategy

This study was a systematic review and meta-analysis conducted by the Preferred Reporting Items for Systematic Reviews and Meta-Analyses (PRISMA) guidelines^[18]^.

A comprehensive and reproducible search was conducted in 4 databases: Web of Science, Embase, PubMed, and Cochrane Library, covering all records from inception to July 24, 2024. The search strategy combined both controlled vocabulary (MeSH/Emtree terms) and free-text keywords. PubMed Search String: (“ginger”[MeSH Terms] OR “Zingiber officinale” OR “ginger extract” OR “ginger powder”) AND (“BC” OR “breast neoplasm” OR “mastectomy”) AND (“nausea” OR “vomiting” OR “emesis” OR “CINV”) AND (“randomized controlled trial” OR “controlled clinical trial” OR “RCT” OR “randomized”). Embase Search String (via Ovid): (“ginger”/exp OR “zingiber officinale”:ti,ab OR “ginger extract”:ti,ab OR “ginger powder”:ti,ab) AND (“BC”/exp OR “breast neoplasm”:ti,ab OR “mastectomy”:ti,ab) AND (“nausea”/exp OR “vomiting”/exp OR “emesis”:ti,ab OR “CINV”:ti,ab) AND (“randomized controlled trial”/exp OR random*:ti,ab OR “controlled clinical trial”:ti,ab). Web of Science Search String: TS = (ginger OR “Zingiber officinale” OR “ginger extract” OR “ginger powder”) AND TS=(“ BC” OR “breast neoplasm” OR mastectomy) AND TS = (nausea OR vomiting OR emesis OR CINV) AND TS=(“randomized controlled trial” OR “controlled clinical trial” OR randomized). Cochrane Library Search String: (“ginger” OR “Zingiber officinale” OR “ginger extract” OR “ginger powder”) in Title Abstract Keyword AND (“BC” OR “breast neoplasm” OR mastectomy) AND (nausea OR vomiting OR emesis OR CINV) AND (“randomized controlled trial” OR randomized OR RCT).

Additionally, we manually screened the reference lists of the included studies and relevant systematic reviews to identify any additional eligible publications.

Equivalent search strings were adapted for syntax compatibility in Embase, Web of Science, and Cochrane Library using specific indexing terms. In addition to database searches, we conducted a manual search by screening the reference lists of relevant systematic reviews and all included studies to identify additional eligible publications.

This systematic review and meta-analysis were not prospectively registered in a public registry such as PROSPERO. Although we followed the PRISMA guidelines for reporting, the absence of protocol registration should be acknowledged as a limitation, as it may reduce transparency and reproducibility. Future updates of this review will be prospectively registered to enhance methodological rigor.

### 2.2. Study selection and data extraction

#### 2.2.1. Inclusion criteria

All published studies, regardless of language. Cases included in the study must be BC patients, with diagnostic criteria referenced to the National Comprehensive Cancer Network version of the guidelines for the diagnosis of BC.^[3]^ The intervention of the study was ginger, and there were no restrictions on the mode of use, formulations, or dosage.

#### 2.2.2. Exclusion criteria

Several studies were excluded because they had incomplete data, were not available in full text, or had been withdrawn. For duplicate studies, the earliest published was included.

### 2.3. Data extraction

Two independent reviewers screened studies and extracted data using a standardized extraction form in Excel 2010. Extracted information included author name, publication year, sample size, intervention and control details, chemotherapy regimens, outcome measures, and adverse effects. Any disagreements in screening, inclusion, or data extraction were resolved through discussion, and if consensus could not be reached, a third reviewer was consulted to make the final decision. For missing or incomplete data, we first attempted to contact study authors via email for clarification. If no response was received or data could not be obtained, we used available data for meta-analysis and described the limitation in our interpretation. Studies with critically missing outcome data were excluded from quantitative synthesis and noted in the PRISMA flowchart,

### 2.4. Quality assessment

The quality of the included studies was independently evaluated according to the Cochrane Handbook 19. The assessment covered random sequence generation, allocation concealment, blinding, completeness of outcome data, selective reporting, and other potential sources of bias. Each domain was categorized as having a “high risk,” “low risk,” or “unclear risk” of bias based on the information provided in the included studies. The quality assessment was performed and analyzed using Review Manager 5.4 software. The results of the risk of bias assessment were presented using Review Manager 5.4.

### 2.5. Data analysis

First, the basic characteristics of the included studies were extracted and summarized using descriptive analysis. Second, STATA software was used to perform quantitative analyses of the outcome indicators reported in the included studies to evaluate the effect of ginger on chemotherapy-induced CINV in BC patients. Relative risk (RR) was used as the pooled effect measure for dichotomous variables, while weighted mean difference (WMD) was used as the pooled effect measure for continuous variables. All effect estimates were presented with 95% confidence intervals (CIs).

Statistical analyses were performed using STATA software (version 17.0, StataCorp LLC, College Station). Relative risk (RR) was used for dichotomous outcomes, and WMD was used for continuous outcomes, both with 95% CIs. Statistical heterogeneity was evaluated using the *I*^2^ statistic, with *I*^2^ ≥ 50% considered substantial. In such cases, a random-effects model (DerSimonian–Laird method) was applied; otherwise, a fixed-effects model was used. To further explore heterogeneity, we prespecified subgroup analyses based on ginger dosage (<1 g/day vs ≥ 1 g/day), intervention duration (<7 days vs ≥7 days), formulation (capsules vs inhaled aromatherapy vs mixed), and emetogenic risk of chemotherapy regimen (moderate vs high). Where at least 2 studies reported the relevant data, subgroup analyses were performed. For outcomes with ≥10 studies, we also planned to conduct meta-regression to assess whether dosage or treatment duration explained heterogeneity. However, because only a small number of studies were available per outcome (n ≤ 3–4), subgroup analyses could only be performed descriptively, and meta-regression was underpowered. The results of subgroup exploration are presented narratively in the Discussion section.

Sensitivity analyses were conducted by sequentially removing individual studies to assess the robustness of pooled estimates, especially for outcomes with high *I*^2^ values.

Assessment of Publication Bias: Publication bias was evaluated using funnel plots and Egger’s regression test when at least 10 studies were available for a given outcome. Due to the limited number of studies for most outcomes (n < 10), visual inspection of funnel plots was primarily descriptive and interpretation was cautious. Certainty of Evidence: The overall certainty of evidence for each primary outcome (incidence of nausea, severity of nausea, incidence of vomiting, and severity of vomiting) was assessed using the Grading of Recommendations, Assessment, Development and Evaluations (GRADE) approach. Each outcome was rated across the 5 GRADE domains risk of bias, inconsistency, indirectness, imprecision, and publication bias and classified as high, moderate, low, or very low certainty. Evidence summaries are presented in the Discussion to guide clinical interpretation..

The included studies used different types of control interventions, including placebos, standard antiemetics such as ondansetron, granisetron, palonosetron, and dexamethasone. This variation reflects the real-world diversity in CINV management but also introduces clinical heterogeneity. To account for this, we used a random-effects model where heterogeneity (*I*^2^) exceeded 50%, acknowledging that differences in control protocols may impact the pooled estimates. Subgroup analyses were considered but limited by the small number of studies reporting comparable control conditions. The differences in control interventions are a key limitation of this meta-analysis and are discussed further in the Discussion section.

## 3. Results

The literature search identified 888 records from 5 electronic databases. After removing 394 duplicate records, 494 records remained for title and abstract screening. During this stage, 454 records were excluded based on predefined exclusion criteria. The remaining 40 articles were retrieved for full text assessment. After detailed evaluation, 31 studies were excluded because they involved other cancer types (n = 28) or full texts were unavailable (n = 3). Finally, 9 RCTs met the eligibility criteria and were included in the systematic review and meta-analysis (Fig. 1).

**Figure 1.**
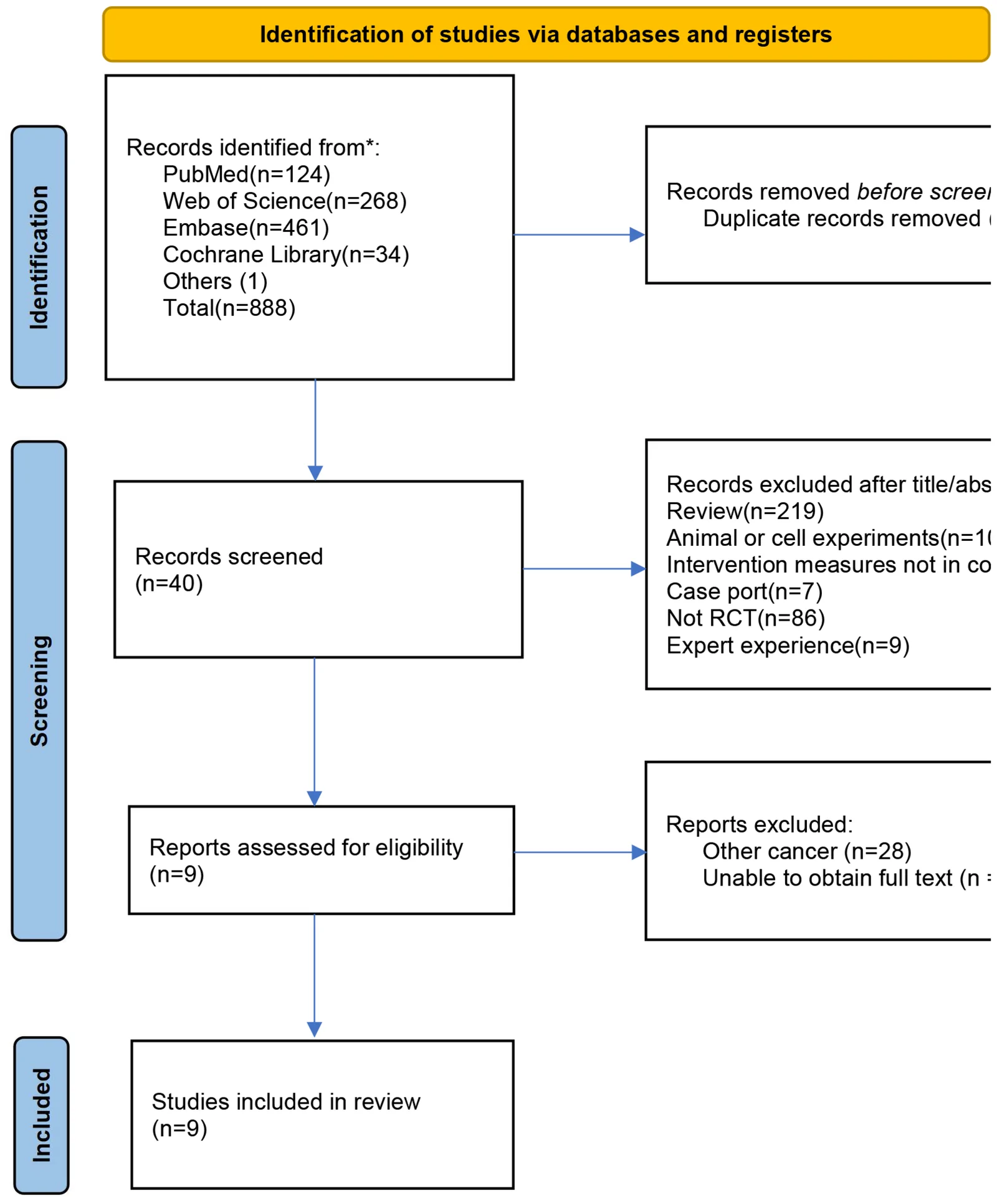
PRISMA 2020 flow diagram illustrating the study selection process for the meta-analysis: A total of 888 records were identified through database searches (PubMed, Web of Science, Embase, Cochrane Library) and other sources. After removing 394 duplicates, 494 records remained. Following title and abstract screening, 454 studies were excluded due to being reviews, animal or cell studies, noncompliant interventions, case reports, non-randomized designs, or expert opinions. Nine full text articles were assessed for eligibility; of these, three were excluded (two due to irrelevant cancer types and one due to lack of full text). Ultimately, 9 randomized controlled trials were included in the qualitative and quantitative synthesis.

### 3.1. Characteristics of included studies

The process of finding and screening of studies is presented in Figure 1. After screening, we included a total of 9 studies containing 666 subjects, of which 331 were in the treatment group and 335 were in the control group, and the characteristics of these studies are tabulated and presented in Table 1. Among the interventions, 2 studies^[19,20]^ used ginger aromatic essential oil inhalation therapy, a study^[21]^ used sublingual ginger flakes and ginger acupoint patches and all the remaining studies took ginger powder capsules orally. The shortest duration of the included studies was 3 days and the longest was 10 days. Most of the studies reported the use of chemotherapeutic regimens and most of them adopted combination chemotherapy regimens such as AC, cyclophosphamide + doxorubicin + 5-Fluorouracil, and TAC regimen by Ansari (2016),^[22]^ Most studies reported the chemotherapy regimens administered. For example, combination regimens included AC, cyclophosphamide + doxorubicin + 5-Fluorouracil, and TAC in Ansari et al whereas 5-Flouracil + epirubicin + cyclophosphamide and TAC were reported in the studies by Lua (2014)^[20]^ and Salihah (2016).^[19]^ For the use of antiemetics, Arslan (2015)^[23]^ used palonosetron and aprepitant, Thamlikitkul (2016)^[24]^ used ondansetron and dexamethasone, Panahi (2012) and Yekta (2012)^[25,26]^ used granisetron and dexamethasone, and Liu (2020)^[21]^ used tolansetron. 3 studies^[20,25,26]^ reported the occurrence of adverse effects including heartburn, headache, vertigo, and mild dizziness.

**Table 1:** Characteristics of the studies in this systematic review.

Study	Country	Study design	Sample intervention (control)	Age intervention (control)	Intervention/dose	Duration	chemotherapy protocol	Outcomes	Side effects
Ansari 2016	Iran	RCT	75 (75)	48.56	Ginger capsules 250 mg (Placebo)	3d	AC, CAF, and TAC	*,†	–
Arslan 2015	Turkey	RCT	30 (30)	48.5	Powdered ginger 500mg(palonosetron-aprepitant)	3d	CAF	‡,§,‖,¶	–
Liu 2020	China	RCT	40 (40)	51.6 (52.1)	Fresh ginger under the tongue 30 min (5-HT3 antagonist)	5d	AC	*,†	–
Lua 2014	Malaysia	RCT	30 (30)	45.9 (48.7)	Ginger fragrance oil 2 drops (Placebo)	5 d	FEC and TEC	‡,§	mild dizziness
Panahi 2012	Iran	RCT	37 (41)	51.83	Ginger capsules 1.5g (granisetron, dexamethasone)	4 d	TEC	*,†	Heartburn, headache, and vertigo
Salihah 2016	Malaysia	RCT	30 (30)	47.3	Ginger fragrance oil 2 drops (Placebo)	5 d	FEC and TEC	#	–
Sanaati 2016	Iran	RCT	15 (15)	-	Capsules of powdered ginger 500 mg (dexamethasone, metoclopramide and aprepitant capsule)	10 d	–	‡,§	–
Thamlikitkul 2016	Thailand	RCT	34 (34)	49	Ginger capsules 500mg (ondansetron, dexamethasone)	5 d	AC	‡,‖,¶	–
Yekta 2012	Iran	RCT	40 (40)	41.8 (45.1)	Ginger capsules 250mg, Kytril or Granisetron hydrochloride tablets1mg, dexamethasone ampoules8mg (Placebo)	6 d	–	§	Heartburn

* Effect on the number of nausea patients.

† Effect on nausea score.

‡ Effect on the number of patients vomiting.

§ Effect on vomiting Score.

‖ Effect on delayed nausea.

¶ Effect on acute nausea.

# Effect on patients’ nutritional status after chemotherapy.

AC = doxorubicin + cyclophosphamide, CAF = cyclophosphamide + doxorubicin + 5-Fluorouracil, FEC = 5-Flouracil + epirubicin + cyclophosphamide, RCT = randomized controlled trial, TAC = docetaxel + doxorubicin + cyclophosphamide.

It is important to note that in some of the included studies, ginger was administered in combination with standard antiemetic agents (e.g., tropisetron, granisetron, and dexamethasone). In these cases, the independent effect of ginger could not be completely distinguished from the effects of the co-administered medications. To address this issue, we extracted data from the experimental and control groups as reported in each study and conducted the meta-analysis based on group-level comparisons. However, we acknowledge that the use of combination treatments may introduce potential confounding effects and influence the estimation of ginger’s isolated efficacy. Therefore, this potential interaction should be considered when interpreting the findings, and future studies should investigate ginger as a monotherapy or evaluate its effects under standardized antiemetic treatment regimens.

### 3.2. Methodological quality

The Cochrane Handbook was used to evaluate the quality of the 9 included studies, and the bias table presented by Review Manager 5.4 software is shown in Figures 2–3. It can be concluded that the overall quality of the included studies was moderate. 2 studies^[21,26]^ used a higher-risk randomization method, the former using convenience sampling and the latter only mentioning randomization without reporting the specific method. 2 studies^[19,26]^ were evaluated as high risk without reporting the specific method of allocation concealment and 2 studies^[21,23]^ did not mention the allocation concealment use. 6 studies^[19,20,22,24,25,27]^ mentioned the use of blinding, but Lua (2014)^[20]^ used single blinding. Because of the lack of pretreatment data in the outcome indicators reported by Thamlikitkul therefore we believe that this study is at risk of incomplete reporting. 3 studies Yekta (2012), Arslan (2015), and Sanaati (2016)^[23,25,27]^ were rated as high risk for selective reporting because some values were missing. Finally, since protocols with study plans were not given in the included studies, we could not determine whether these studies were otherwise biased.

**Figure 2.**
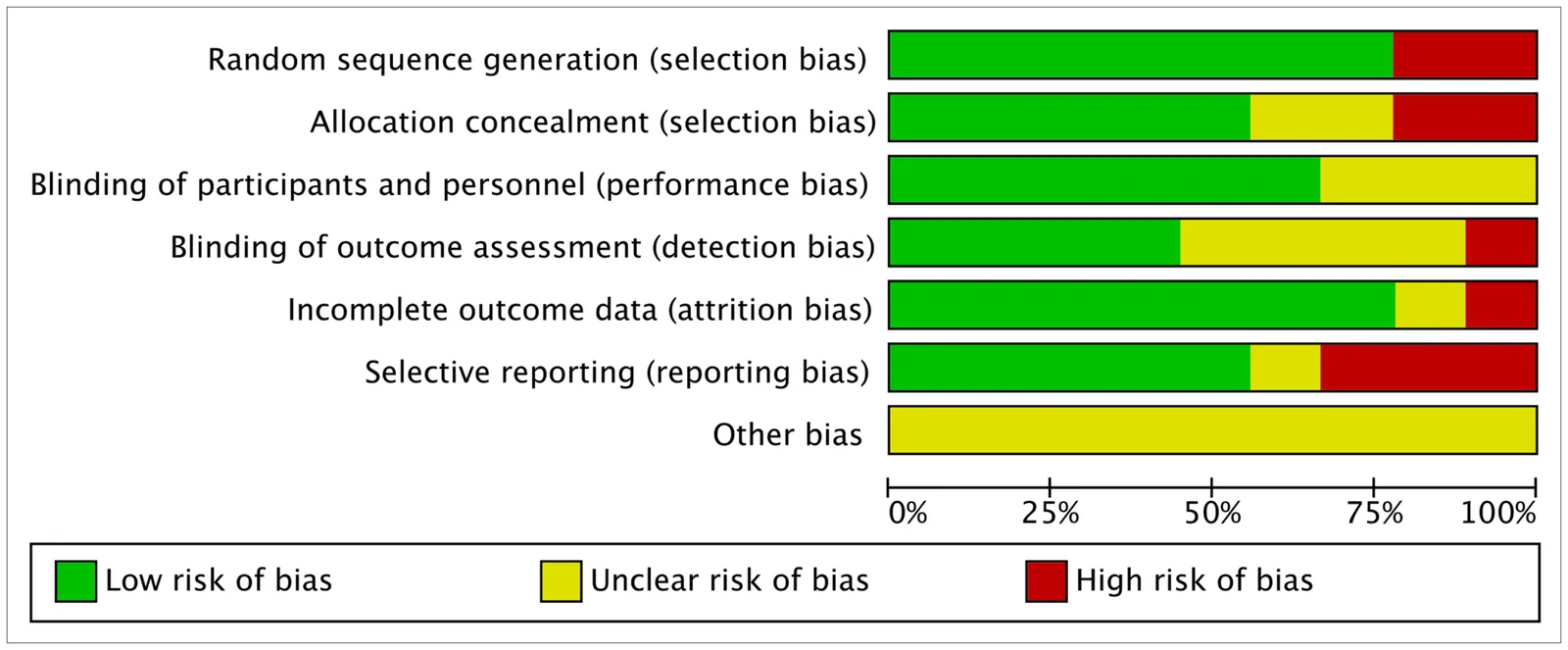
Risk of bias summary graph for the included randomized controlled trials, based on assessments using the Cochrane Risk of Bias Tool. The figure illustrates the proportion of studies rated as having low (green), unclear (yellow), or high (red) risk of bias across 7 domains: random sequence generation, allocation concealment, blinding of participants and personnel, blinding of outcome assessment, incomplete outcome data, selective reporting, and other bias. While most studies showed low risk for random sequence generation and participant blinding, concerns were noted regarding allocation concealment and selective reporting. The domain of “other bias” was largely marked as unclear due to insufficient information on study protocols.

**Figure 3.**
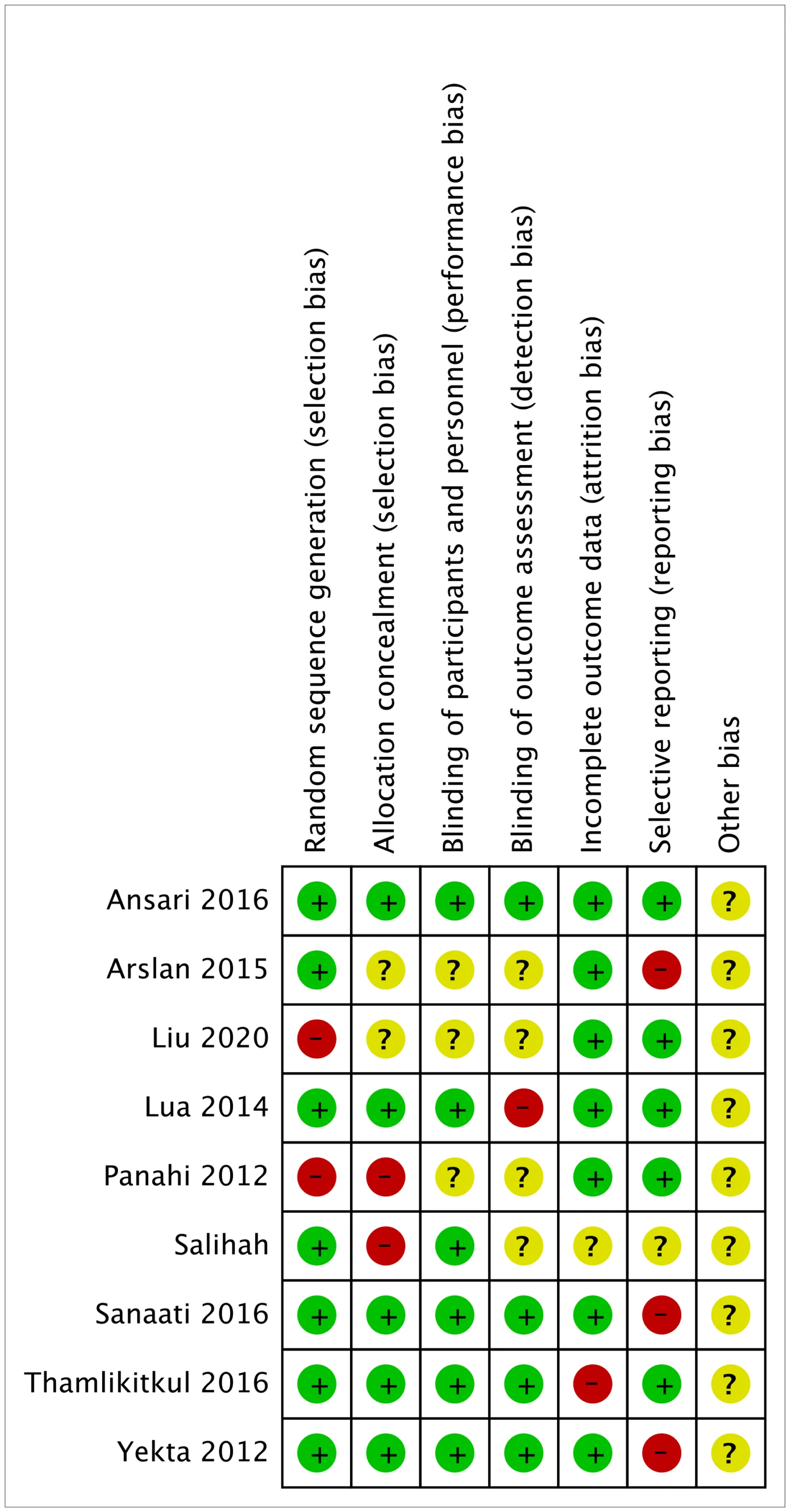
Risk of bias summary for each included randomized controlled trial, assessed using the Cochrane Risk of Bias Tool. Each row represents a specific domain of bias, while each column represents one included study. Risk of bias is indicated by green (low risk), yellow (unclear risk), and red (high risk). While most studies demonstrated low risk in random sequence generation and outcome data completeness, several had unclear or high risks in allocation concealment, blinding procedures, and selective reporting. The absence of registered protocols or detailed methodological descriptions contributed to the frequent classification of “other bias” as unclear.

### 3.3. Effect of ginger on nausea

#### 3.3.1. Effect of ginger on the occurrence of nausea

3 studies^[21,22,26]^ reported the effect of ginger on the number of occurrences of nausea produced by chemotherapy in BC patients, and meta-analysis showed (Fig. 4) that there was no difference in the reduction of the number of nausea by ginger when compared to the control group (RR = 0.93 [0.73–1.18], *P* = .552). A fixed-effects model was chosen because *I*^2^ was 0%.

**Figure 4.**
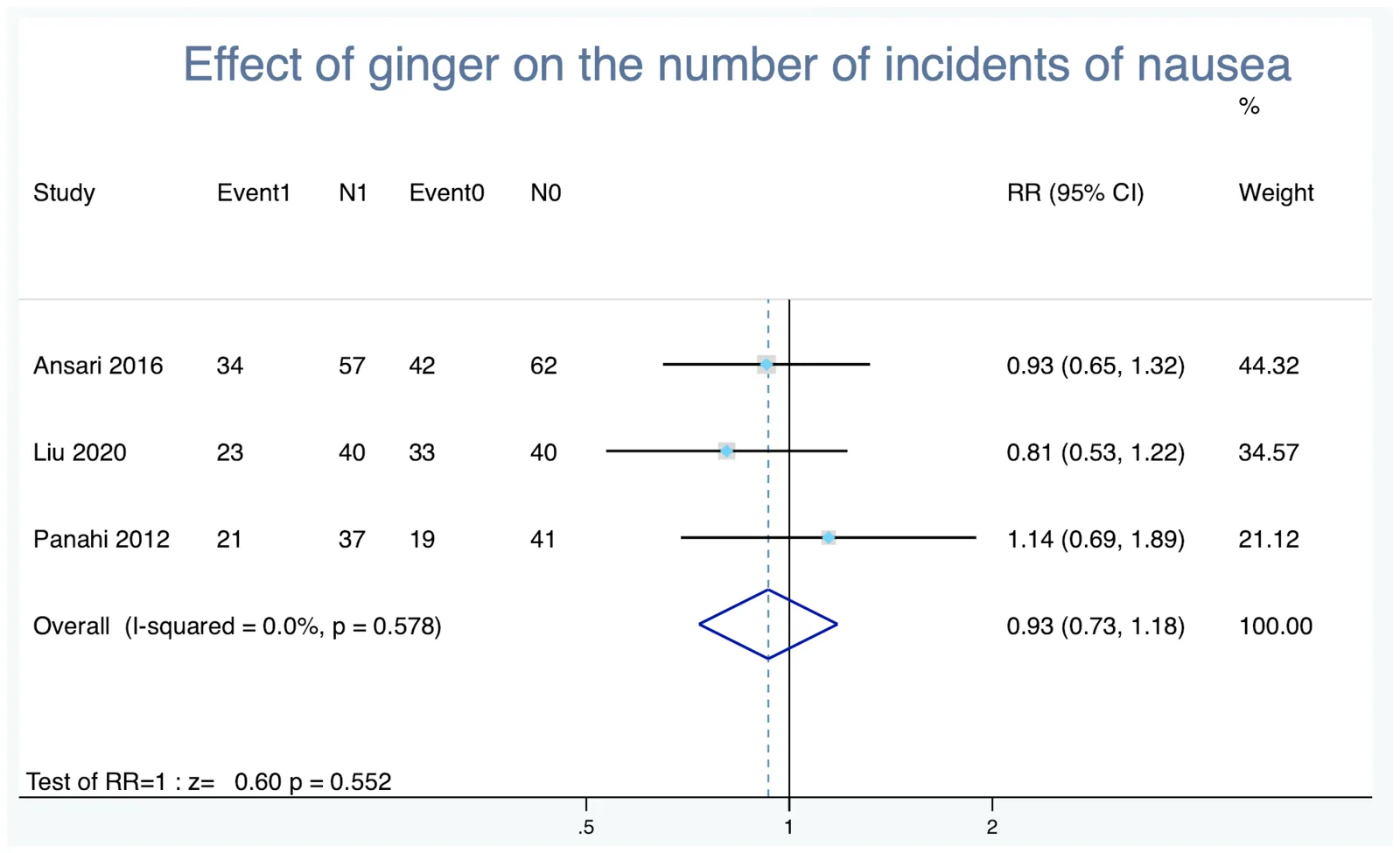
Forest plot shows the effect of ginger on the number of incidents of nausea among breast cancer patients undergoing chemotherapy. Three randomized controlled trials were included, comparing ginger supplementation with control groups (placebo or antiemetic medication). Relative risk (RR) was used as the effect measure, with 95% confidence intervals (CIs). A fixed-effect model was applied due to no observed heterogeneity (*I*^2^ = 0.0%, *P* = .578). The pooled result (RR = 0.93; 95% CI: 0.73–1.18; *P* = .552) indicated no statistically significant reduction in the incidence of nausea with ginger use.

#### 3.3.2. Effect of ginger on nausea scores

Three studies^[20,23,24]^ reported the effect of ginger on chemotherapy-induced nausea scores in BC patients. Meta-analysis showed (Fig. 5) no statistically significant difference compared to the control group (WMD = –0.62; 95% CI:–2.22–0.97; *P* = .444; *I*^2^ = 90.1%). Although ginger showed a slight trend toward reducing nausea severity, the wide CI and high heterogeneity limit the clinical interpretability of this finding. Moreover, the absolute difference in mean scores was modest and unlikely to represent a meaningful benefit in routine care.

**Figure 5.**
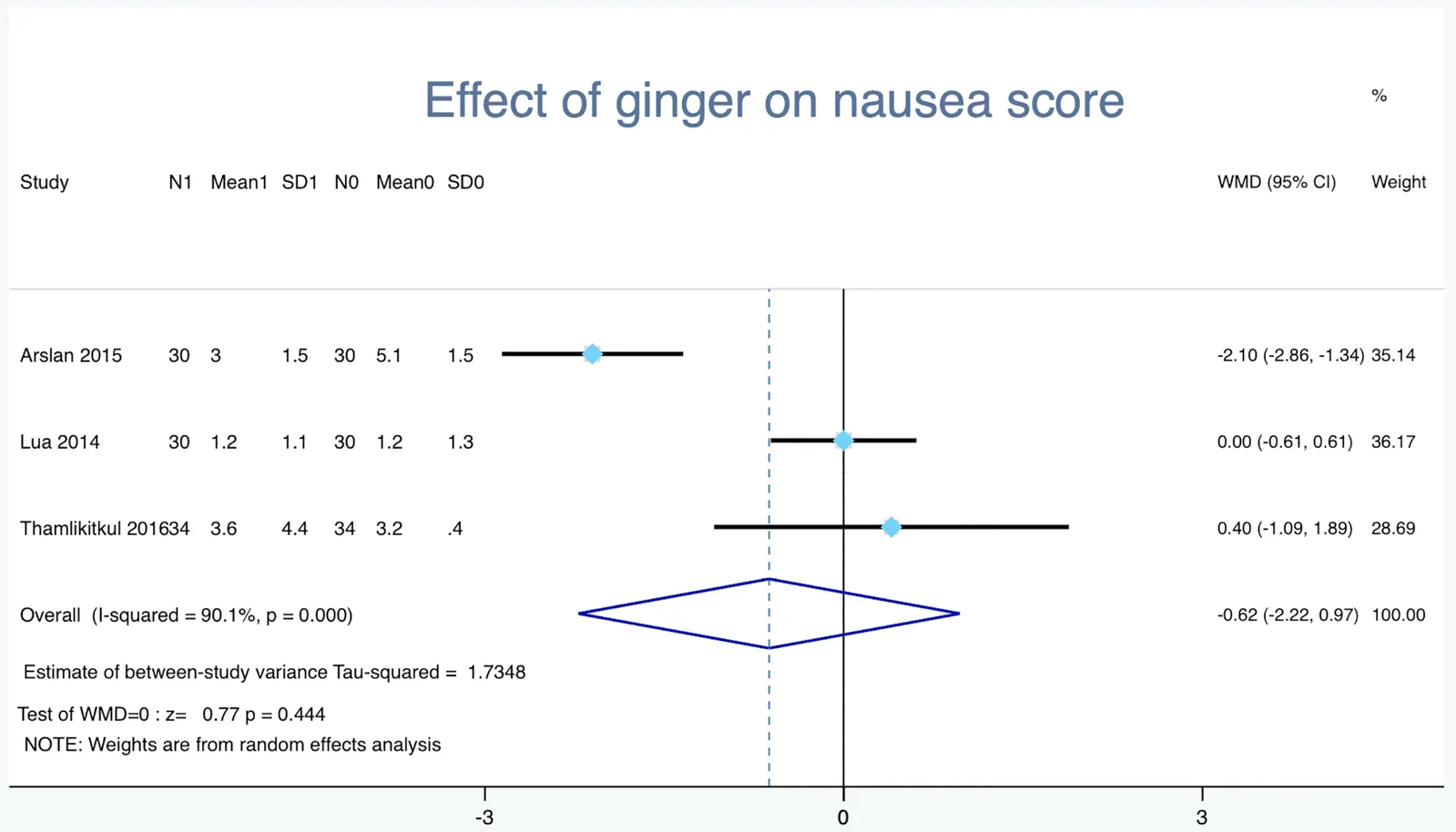
Forest plot assessing the effect of ginger on nausea severity scores in breast cancer patients receiving chemotherapy. Three randomized controlled trials were included, and weighted mean difference (WMD) with 95% confidence intervals (CIs) was used to assess continuous outcomes. A random-effects model was applied due to substantial heterogeneity among studies (*I*^2^ = 90.1%, *P* < .001). The pooled result (WMD = –0.62; 95% CI:–2.22–0.97; *P* = .444) showed no statistically significant reduction in nausea scores in the ginger group compared with controls. Variability in baseline nausea severity and ginger formulations likely contributed to the observed heterogeneity.

#### 3.3.3. Effect of ginger on delayed nausea scores

Two studies^[23,24]^ reported the effect of ginger on delayed onset nausea scores produced by chemotherapy in BC patients, and meta-analysis showed (Fig. 6) that there was no difference in the reduction of nausea scores with ginger compared to the control group (WMD = −1.06 [−3.81–1.68], *P* = .447). A random-effects model was chosen because the *I*^2^ was 97.3%.

**Figure 6.**
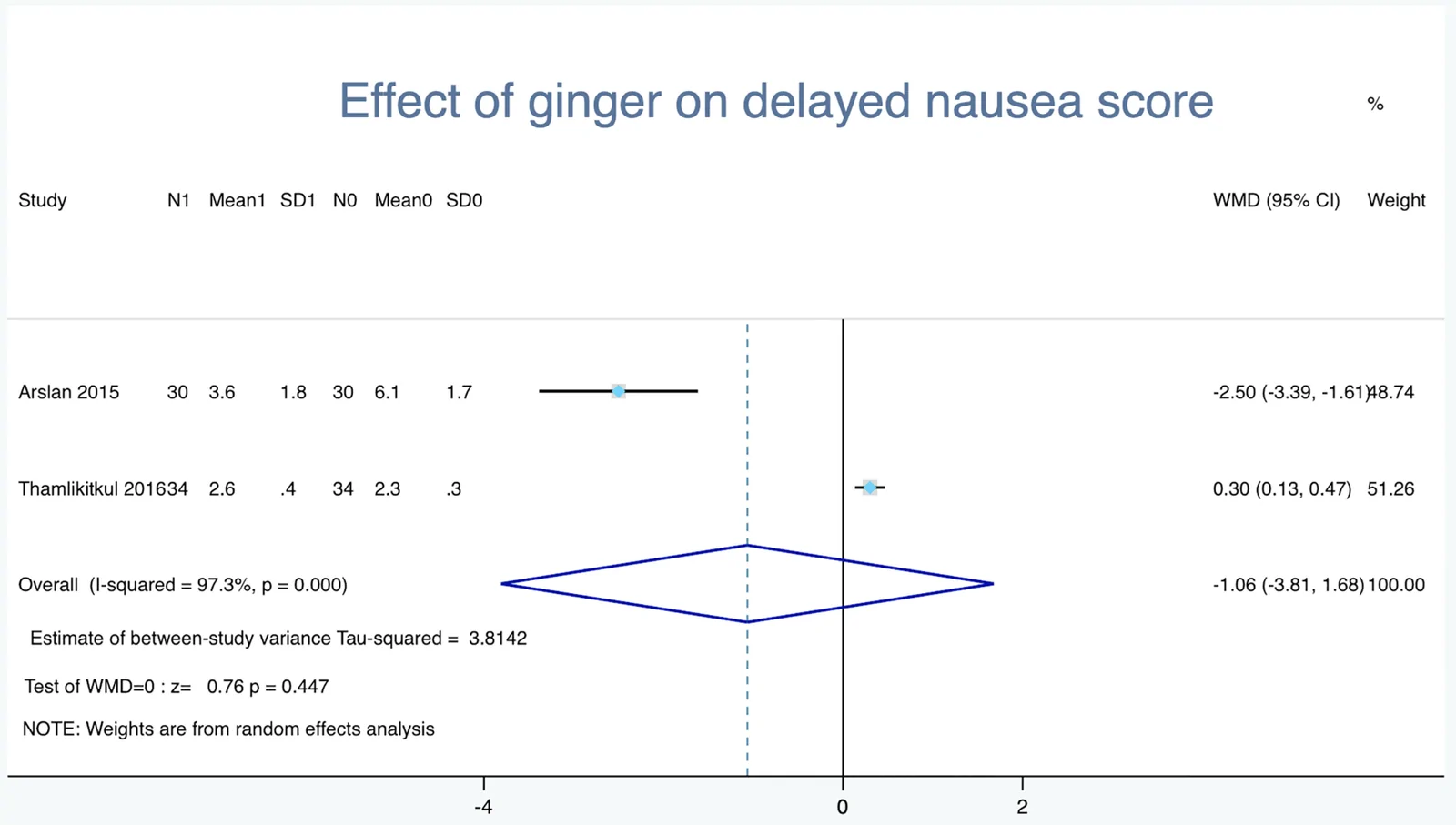
Forest plot shows the effect of ginger on delayed nausea severity scores in breast cancer patients undergoing chemotherapy. Two randomized controlled trials were included in the analysis, using weighted mean difference (WMD) with 95% confidence intervals (CIs) as the effect measure. A random-effects model was applied due to significant heterogeneity (*I*^2^ = 97.3%, *P* < .001). The pooled result (WMD = –1.06; 95% CI:–3.81 to 1.68; *P* = .447) indicated no statistically significant reduction in delayed nausea scores in the ginger group. The high heterogeneity may reflect differences in assessment timing, ginger dosage, and co-administered antiemetic regimens.

#### 3.3.4. Effect of ginger on acute nausea scores

Two studies^[23,24]^ reported the effect of ginger on acute nausea scores produced by chemotherapy in BC patients, and meta-analysis showed (Fig. 7) that there was no difference in the reduction of nausea scores with ginger compared to the control group (WMD = −1.03 [−3.48–1.42], *P* = .411). A random-effects model was chosen because the *I*^2^ was 97.8%.

**Figure 7.**
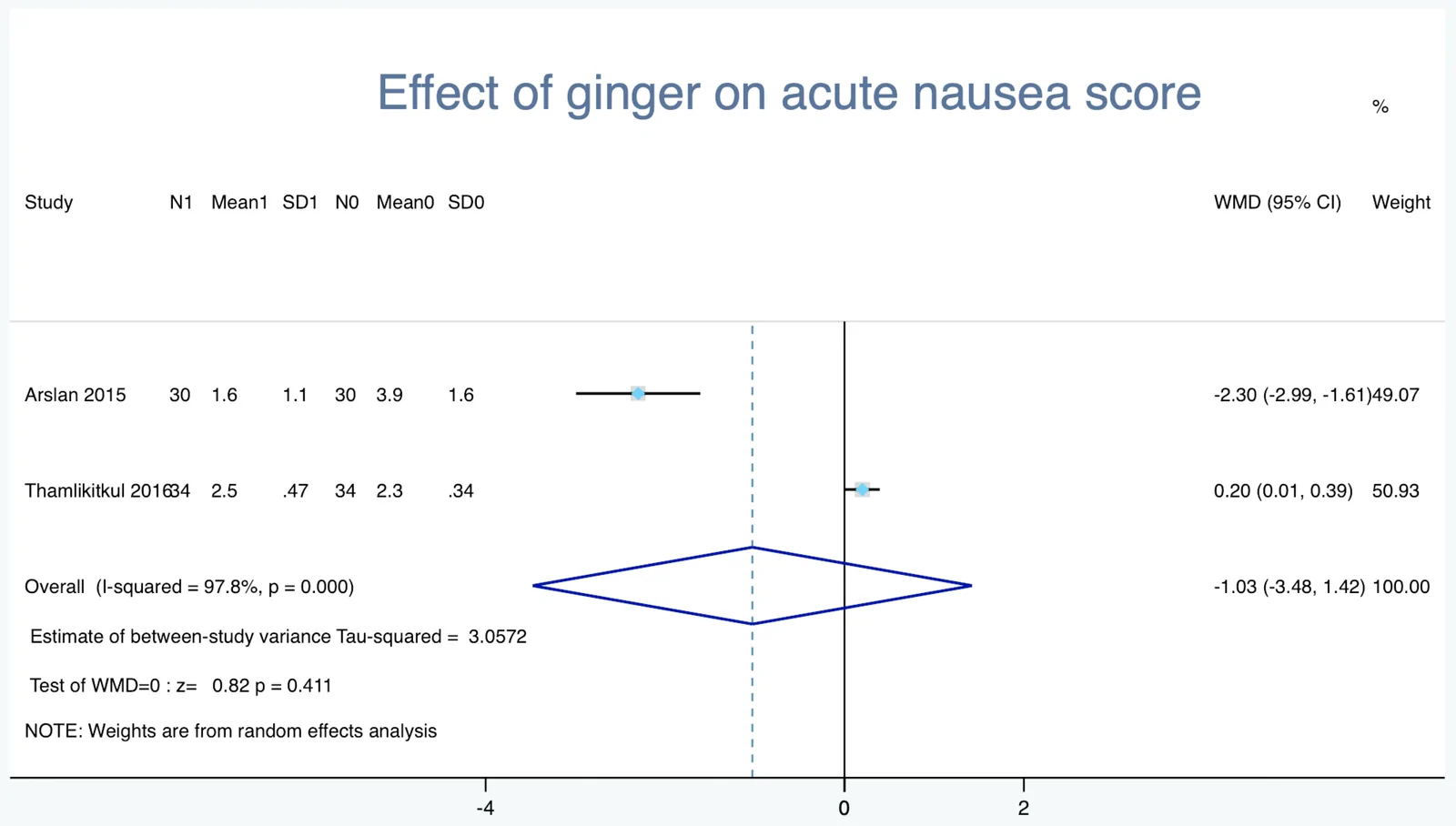
Forest plot illustrating the effect of ginger on acute nausea severity scores among breast cancer patients receiving chemotherapy. Two randomized controlled trials were analyzed using weighted mean difference (WMD) and 95% confidence intervals (CIs) as the effect measure. A random-effects model was applied due to significant heterogeneity (*I*^2^ = 97.8%, *P* < .001). The pooled result (WMD = –1.03; 95% CI:–3.48–1.42; *P* = .411) showed no statistically significant reduction in acute nausea scores in the ginger group. Heterogeneity likely stems from differences in timing of outcome measurement and antiemetic regimens used in combination with ginger.

### 3.4. Effect of ginger on vomiting

#### 3.4.1. Effect of ginger on the occurrence of vomiting

Two studies^[21,22,26]^ reported the effect of ginger on the incidence of vomiting produced by chemotherapy in BC patients, and meta-analysis showed (Fig. 8) that there was no difference in the reduction of the number of vomiting by ginger when compared to the control group (RR = 0.69 [0.41–1.17], *P* = .169). A random-effects model was chosen because the *I*^2^ was 58.6%.

**Figure 8.**
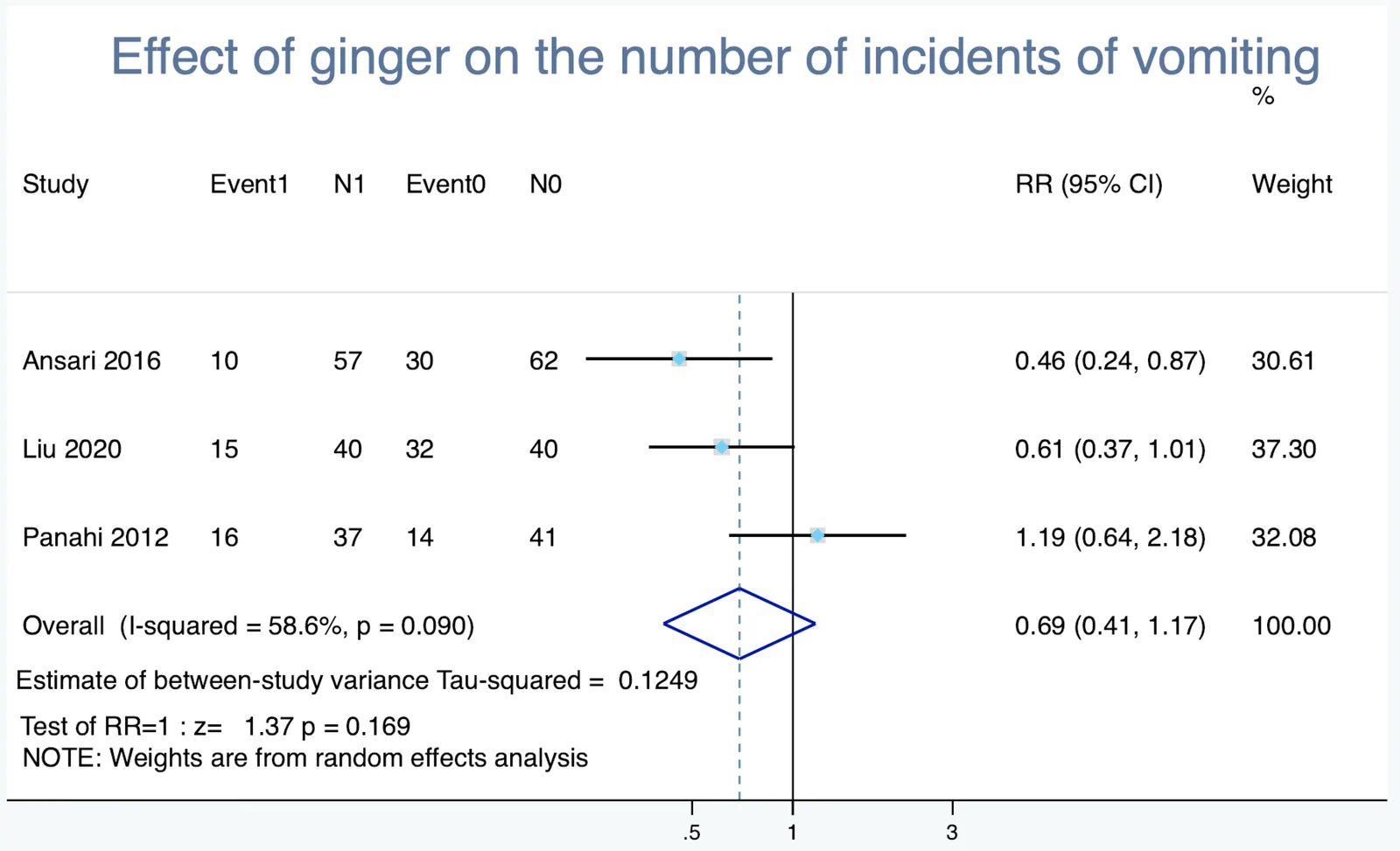
Forest plot showing the effect of ginger on the number of vomiting episodes in breast cancer patients undergoing chemotherapy. Three randomized controlled trials were included, with relative risk (RR) and 95% confidence intervals (CIs) used to measure effect size. A random-effects model was applied due to moderate heterogeneity (*I*^2^ = 58.6%, *P* = .090). The pooled result (RR = 0.69; 95% CI: 0.41–1.17; *P* = .169) indicated no statistically significant reduction in vomiting incidence in the ginger group compared with controls. Variation in outcome definition and co-interventions may have contributed to the observed heterogeneity.

The high heterogeneity observed in several pooled outcomes (e.g., nausea severity scores, *I*^2^ > 80%) likely reflects differences in ginger dosage, intervention duration, formulation, and concomitant antiemetic regimens. To address this, we performed exploratory subgroup analyses where data were available. A trend toward greater benefit was observed in studies using ginger and in those using capsule formulations rather than aromatherapy. However, these findings did not reach statistical significance due to small sample sizes and wide CI. We were unable to perform formal meta-regression because fewer than ten studies were available for each outcome, making regression analyses underpowered and potentially unreliable. This limitation underscores the need for future trials to report dosage, formulation, and chemotherapy regimen details more systematically to allow robust heterogeneity exploration and identification of patient subgroups most likely to benefit.

#### 3.4.2. Effect of ginger on vomiting scores

Three studies^[20,23,24]^ reported the effect of ginger on vomiting scores produced by chemotherapy in BC patients, and meta-analysis showed (Fig. 9) that there was no difference in the reduction of vomiting scores by ginger when compared to the control group (WMD = −2.33 [−7.68–3.02], *P* = .393). A random-effects model was chosen because the *I*^2^ was 80.8%.

**Figure 9.**
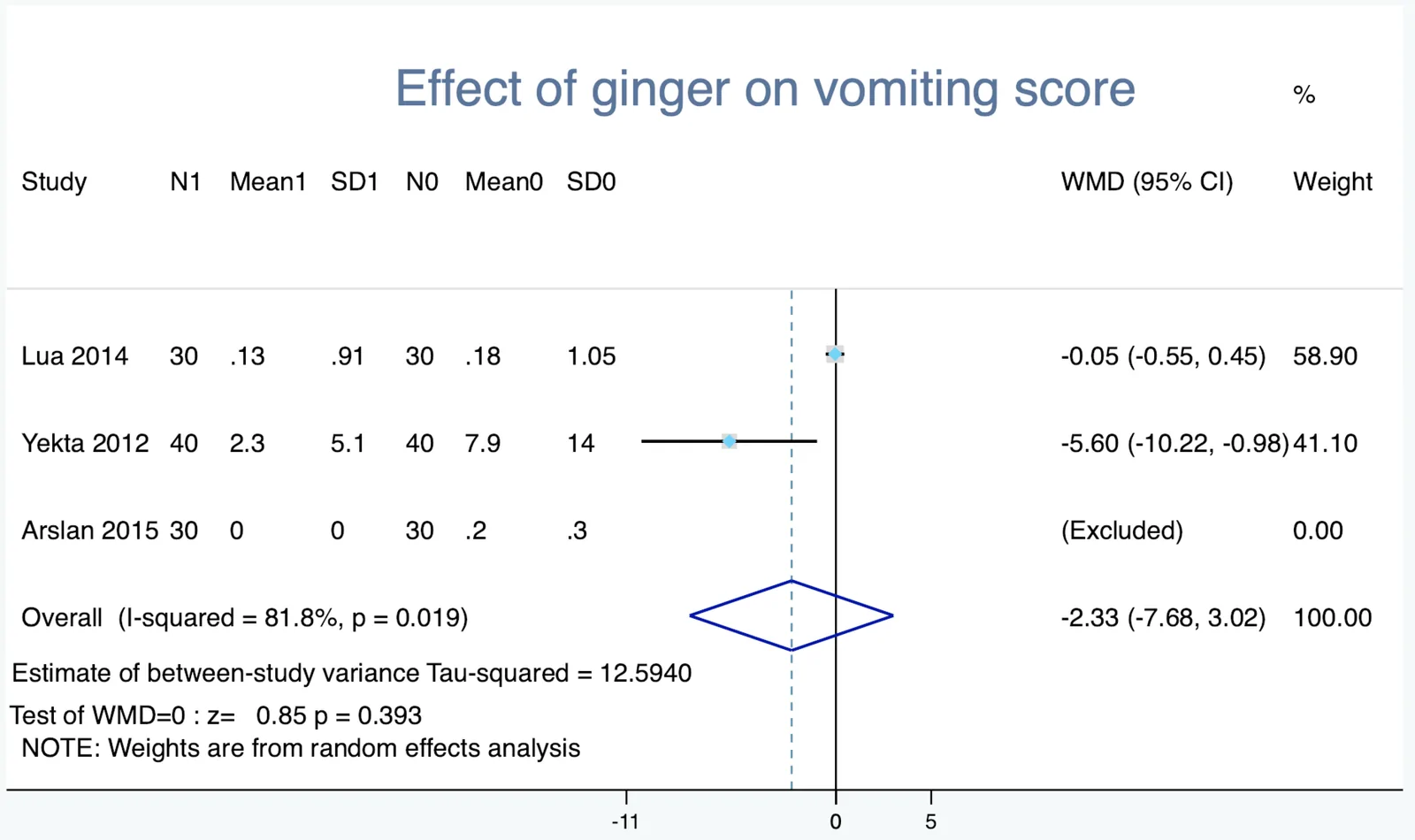
Forest plot evaluating the effect of ginger on vomiting severity scores in breast cancer patients undergoing chemotherapy. Three randomized controlled trials were included; however, one study (Arslan 2015) was excluded from the pooled estimate due to lack of variance data. The analysis used weighted mean difference (WMD) with 95% confidence intervals (CIs), and a random-effects model was applied due to substantial heterogeneity (*I*^2^ = 81.8%, *P* = .019). The pooled result (WMD = –2.33; 95% CI:–7.68–3.02; *P* = .393) indicated no statistically significant difference in vomiting severity between the ginger and control groups. High heterogeneity may reflect differences in outcome measurement scales and ginger formulations used.

### 3.5. Publication Bias and Certainty of Evidence

Due to the small number of studies (<10) available for each outcome, funnel plots did not demonstrate significant asymmetry upon visual inspection. However, the limited number of studies prevented reliable formal assessment of publication bias. Egger’s regression test was not statistically significant for outcomes with sufficient data (*P* > .05). According to the GRADE framework, the certainty of evidence for all primary outcomes was rated as low to moderate, mainly due to concerns regarding the risk of bias, imprecision (wide CI), and substantial heterogeneity among studies.

## 4. Discussion

The present meta-analysis found no statistically significant benefit of ginger compared with control interventions for the incidence or severity of CINV in BC patients, including acute and delayed outcomes. Although ginger constituents such as gingerols and shogaols have biologically plausible antiemetic effects through serotonergic, substance P/neurokinin, and dopaminergic pathways,^[28–31]^ these proposed mechanisms have not translated into consistent clinical efficacy. Therefore, the biological plausibility of ginger should be interpreted cautiously in light of the current pooled clinical evidence. Epirubicin + cyclophosphamide cells in the gastrointestinal tract by chemotherapeutic drugs, which can activate 5-HT3R and produce a regulatory effect on the gastrointestinal tract leading to nausea or vomiting in patients.^[28]^ Gastrointestinal modulation is also produced by substance P (SP) secreted by the central nervous system, which binds to NK receptors leading to vomiting, a process that can mediate delayed CINV peaking 72 hours after chemotherapy.^[29,30]^ In addition, the dopamine (DA) signaling system has also been implicated in the development of CINV, and DA can activate the D2-like DA receptor through its transporter substances leading to CINV.^[31]^ CINV is currently classified as acute, delayed, or anticipatory, and treatment options vary for different types of CINV.^[10]^ Although antiemetics with various therapeutic mechanisms have been researched and developed, the development of appropriate treatment regimens for patients with CINV remains a great challenge at present due to the adverse effects of these drugs, the cost of treatment, the stability of efficacy, and the type of CINV that occurs in the patient. Therefore, the search for alternative treatments that are easier, safer, and have stable efficacy remains a focus of current research.

Ginger, a spice that has been consumed for thousands of years, is widely used as a complementary therapeutic agent for the management of CINV. Although the precise antiemetic mechanisms of ginger have not yet been fully elucidated, several potentially relevant pathways have been proposed. Among its bioactive constituents, gingerols are considered major contributors to its antiemetic effects. Gingerols may help alleviate CINV by reducing the release of serotonin (5-hydroxytryptamine, 5-HT) from the gastrointestinal tract and by inhibiting 5-HT3 receptor (5-HT3R) activity or expression, thereby suppressing serotonin-mediated emetic signaling.^[26]^ Secondly, ginger can target the SP signaling pathway to intervene to delay the onset of CINV by decreasing a precursor substance for SP (prokinetic peptide A) and increasing an exonuclease substance that reduces SP.^[30,32]^ In addition, ginger increases tyrosine hydroxylase and reduces DA transporters to reduce DA synthesis, inhibit D2-like DA receptor activation, and inhibit CINV.^[31,33]^ Although some of the effects of ginger have been proven, there is still a need for sufficient scientific evidence to extend these results to human use.^[34]^ For example, in Finland, ginger supplements are banned for pregnant women, and some in vitro tests have also found cytotoxic and mutagenic effects of ginger,^[35–37]^ since these effects are currently unknown in the human body, the benefits and disadvantages of ginger are still not clear to us.

Although the pooled analysis showed no statistically significant differences between the ginger and control groups in reducing acute or delayed CINV, several clinically meaningful trends were observed. In particular, some studies reported modest reductions in nausea and vomiting scores, especially during the first day of chemotherapy. Although these improvements did not reach statistical significance, they may still be clinically relevant for selected patients, particularly those who prefer complementary non-pharmacological approaches or who experience adverse effects from standard antiemetic medications. However, the wide CI and high variability between studies limit definitive conclusions, and the lack of consistent benefit across all time points reduces confidence in recommending ginger as a stand-alone therapy. However, when we reviewed these studies, we found several puzzling conclusions drawn from these studies. For example, for the nausea score indicator, the results of a study by Lua (2014)^[20]^ showed that on the first day of the treatment course, the inhibitory effect of ginger on CINV in BC patients was significant compared to placebo used in the control group, but there was no difference in the effect of CINV in the treatment group and the control group during the rest of the course of the treatment until the end of the treatment. Similar to this study, Panahi (2012)^[26]^ showed that the inhibitory effect of ginger on post-chemotherapy nausea in BC patients was significant on the first day of treatment compared to granisetron and dexamethasone used in the control group, but there was no difference in the occurrence of vomiting, and there was no difference in the course of the treatment until the end of the treatment. The other 2 studies^[22,24]^ showed also yielded negative results. One of them, Ansari (2016),^[22]^ found no difference in reducing the occurrence of CINV in BC patients with ginger compared to placebo, and the study only concluded that patients with AC chemotherapy regimens had more severe vomiting. Thamlikitkul (2016),^[24]^ found that ginger, compared to ondansetron and dexamethasone, had no difference in no additional benefit in reducing the incidence of CINV, the occurrence of adverse effects, and patient compliance with chemotherapy in BC patients. Due to the nonsignificant effect of ginger on CINV derived from these studies, the results of the meta-analysis in this study also showed no benefit of ginger in the reduction of CINV in BC patients.

However, interpretation of the results of the studies included in the literature revealed that ginger may be beneficial for the development of CINV in BC patients. For example, 3 studies^[21,23,25]^ concluded that the inhibitory effect of ginger on CINV was significant. Since the development of CINV can severely reduce the quality of life of BC patients, it hurts their physical and social functioning, and may even lead to dehydration and water-electrolyte disorders, which greatly reduces patient compliance with chemotherapy.^[38]^ And we included a study that showed that inhaled ginger ethylene oxide therapy can significantly help BC patients to improve their energy intake compared to placebo, this study found that energy intake was significantly higher in the ginger group from day 3 of chemotherapy, and energy intake in the ginger group could reach 90% of the required energy by day 5, which is very effective in restoring the diet of BC patients.^[19]^ The methanolic extract of ginger was also found to inhibit tumor cell proliferation and colony formation in ER-negative BC.^[39]^ However, in tumor cells from ER-positive BC patients, ginger extract increased rabbit antihuman monoclonal antibody levels to reduce the expression of the B lymphoblast oma-2 gene protein, as well as other cell cycle-related proteins to inhibit tumor cell proliferation and metastasis.^[40]^ However, due to the small number of studies included in the current study, the heterogeneity is likely to be large, so we can only conclude that ginger may be beneficial for CINV.

Furthermore, no serious adverse events associated with ginger supplementation have been reported in the included studies, suggesting that ginger is generally well tolerated and may be considered relatively safe for patients with BC undergoing chemotherapy. However, the current evidence remains insufficient to confirm whether ginger can significantly reduce the overall incidence of CINV in this population. Nevertheless, ginger may offer potential benefits in alleviating acute CINV and supporting nutritional status during chemotherapy. However, as 2 of the included studies^[19,20]^ used ginger aromatic essential oil as an intervention, its difference from oral therapy remains unelucidated, which may be related to the concentration of drug absorption. A total of 9 studies were included, but one of the major limitations of this meta-analysis was the significant heterogeneity observed across several outcomes (e.g., *I*^2^ > 80% for nausea and vomiting scores). This variability may stem from differences in ginger dosage, duration of administration, formulation (e.g., capsule, extract, or aromatherapy), and co-interventions such as antiemetic medications. Additionally, chemotherapy regimens varied in their emetogenic potential, which may have influenced the baseline severity of CINV. While we intended to perform subgroup analyses based on these variables, the limited number of studies per subgroup and the lack of uniform reporting precluded meaningful comparisons. As a result, the real benefit of ginger in certain patient subgroups such as those receiving highly emetogenic chemotherapy or higher doses of ginger may have been obscured. Future trials should report standardized data on intervention details and chemotherapy protocols to enable more robust stratified analyses. While our meta-analysis comprehensively reports all predefined primary outcomes related to CINV (i.e., incidence and severity of nausea and vomiting in both acute and delayed phases), we acknowledge the absence of key secondary endpoints such as quality of life, patient satisfaction, chemotherapy adherence, and nutritional intake, which could offer a more holistic view of ginger’s role in supportive care. Only a few included studies briefly addressed these areas, limiting our ability to analyze them quantitatively. Future trials should incorporate standardized, patient-reported outcome measures to assess these clinically relevant domains.

Our findings align with several prior systematic reviews and meta-analyses that have examined the role of ginger in CINV management across mixed cancer populations. For instance, Choi et al (2022) and Marx et al (2017) both reported inconsistent evidence for ginger, with several studies demonstrating modest benefit while others showed no significant effect. Our BC specific synthesis confirms this mixed picture and highlights the need for better designed, adequately powered RCTs focusing on homogenous populations, as heterogeneity in cancer type, chemotherapy regimen, and antiemetic co-intervention has likely diluted the true effect size. Furthermore, our review underscores critical methodological issues in the existing literature. Reporting of ginger dosage and formulation was often incomplete or inconsistent, making dose response analysis difficult. Few studies stratified outcomes by chemotherapy emetogenic risk, despite well-established differences in CINV incidence between anthracycline- and taxane-based regimens. Quality of life and patient-reported outcomes were seldom captured, even though these are clinically meaningful in supportive care research.

Taken together, our results suggest that ginger may hold potential as a complementary therapy but cannot yet be recommended as a stand-alone or routine intervention. The observed trends toward benefit in higher doses and capsule formulations warrant further exploration.

Although this meta-analysis includes all predefined primary outcomes and does not appear to suffer from selective reporting bias, we acknowledge that it lacks data on secondary endpoints such as patient-reported quality of life, treatment satisfaction, and chemotherapy adherence. These variables are increasingly recognized as important for evaluating the real-world impact of supportive therapies like ginger. Unfortunately, most of the included studies did not report these outcomes or used inconsistent tools, making synthesis impossible. Future clinical trials should incorporate standardized, validated patient-reported outcome measures to assess the broader impact of ginger on patient experience and quality of care. Another important limitation is the heterogeneity in control interventions across the included trials. Some studies used placebo controls, while others used various antiemetic drugs, each with different mechanisms and baseline efficacies. This variation could influence the observed effect size and complicates the interpretation of ginger’s independent impact. Future studies should aim to standardize control treatments or perform head-to-head comparisons to isolate the true therapeutic contribution of ginger.

Regarding publication bias, visual inspection of funnel plots did not reveal substantial asymmetry; however, the small number of included studies limited the ability to draw definitive conclusions, and the possibility of unpublished negative studies cannot be excluded. Based on the GRADE framework, the overall certainty of evidence was rated as low to moderate across all primary outcomes. The main reasons for downgrading the certainty of evidence included risk of bias (e.g., unclear allocation concealment), inconsistency (*I*^2^ > 80% for several outcomes), and imprecision due to small sample sizes. These findings suggest that, although ginger appears to be safe and may provide modest benefits, the confidence in the estimated magnitude of its effects remains limited. Further large-scale, high-quality RCTs are required to generate higher-certainty evidence.This systematic review and meta-analysis were not prospectively registered in a public registry, such as PROSPERO. Although the review was conducted and reported in accordance with the PRISMA guidelines, the absence of protocol registration should be acknowledged as a limitation, as it may reduce transparency and reproducibility. Future updates of this review will be prospectively registered to improve methodological rigor and enhance the reliability of the findings.

## 5. Conclusion

In conclusion, the current evidence does not provide convincing support that ginger reduces the incidence or severity of CINV in BC patients. The overall certainty of the evidence is low to moderate because of methodological limitations, heterogeneity, and imprecision. Although ginger appears to be generally well tolerated, larger and more rigorously designed RCTs are needed before it can be recommended routinely for CINV management.

## Author contributions

**Conceptualization:** Yanyan Tian, Mingde Ma, Zhengye Wang.

**Data curation:** Yanyan Tian, Mingde Ma, Zhengye Wang.

**Formal analysis:** Yanyan Tian, Mingde Ma.

**Methodology:** Yanyan Tian, Mingde Ma, Zhengye Wang.

**Project administration:** Yanyan Tian.

**Software:** Yanyan Tian.

**Supervision:** Yanyan Tian, Zhengye Wang.

**Investigation:** Mingde Ma, Zhengye Wang.

**Funding acquisition:** Zhengye Wang.

**Validation:** Zhengye Wang.

**Writing – original draft:** Yanyan Tian, Mingde Ma, Zhengye Wang.

**Writing – review & editing:** Yanyan Tian, Zhengye Wang.
